# Supplementary material for: Visual transformer and deep CNN prediction of high-risk COVID-19 infected patients using fusion of CT images and clinical data
Source: BMC Med Inform Decis Mak. 2023 Nov 17;23:265. doi: 10.1186/s12911-023-02344-8 (PMC10656999; doi:10.1186/s12911-023-02344-8)
Supplement: Supplementary file 1 — Additional file 1: Appendix A. Table A.1. Patients’ clinical measurements (mean±std). X(Y%): X represents the label counts in the population, and the associated% of the label in the population is shown with Y. Italic labels represent the excluded labels previouly explained in 'Clinical data trimming' section. Appendix B. Figure B.1. The suggested set of 30 selected clinical labels from ExtraTree classifier. Figure B.2. The suggested set of 13 clinical labels from SelectKbest algorithm. Appendix C. Table C.1. Selected hyperparameters from the Genetic Algorithm for the 3D-CNN CT model ('3D-CNN CT model' section) and the medial 3D-CNN fusion model ('3D‑CNN models on fusion data' section). Appendix D. Table D.2. Results of the seven conventional algorithms in 'pre-processing of clinical data' section on the 13 selected clinical labels from SelectKBest algorithm. Table D.3. Results of the seven conventional algorithms in 'pre-processing of clinical data' section on the 30 selected clinical labels from ExtraTree classifier. Table D.4. Results of the seven conventional algorithms in '3D‑CNN models on fusion data' section on the 25 extracted features from PCA algorithm. [file 12911_2023_2344_MOESM1_ESM.docx]

# Supplementary Information

# Appendix A

Table A.1. Patients’ clinical measurements (mean±std). X(Y%): X represents the label counts in the population, and the associated % of the label in the population is shown with Y. *Italic labels* represent the excluded labels previouly explained in section 3.2.1.1.

| **Clinical variable/label** | **Total (n=380)** |
| --- | --- |
| **Gender** | |
| Female | 133(35%) |
| Male | 247(65%) |
| **Age** | |
| Mean ± SD | 53.82±17.92 |
| **Exposure history** | |
| EHF ^*^ | 57(15%) |
| HOT ^*^ | 29(7.63%) |
| ESP ^*^ | 83(21.84%) |
| CIH ^*^ | 50(13.16%) |
| **Comorbid disease** | |
| Diabetes Mellitus | 77(20.26%) |
| Vascular disease | 59(15.53%) |
| Obesity | 49(12.89%) |
| Smoking | 24(6.32%) |
| Kidney | 19(5.00%) |
| Asthma | 13(3.42%) |
| Other lung diseases | 23(6.05%) |
| Malignancy | 12(3.16%) |
| Hematic | 10(2.63%) |
| Rheumatology | 10(2.63%) |
| Neurological | 14(3.68%) |
| Hypertension | 47(12.37%) |
| UCC^*^ | 12(3.16%) |
| **Clinical manifestations** | |
| Fever | 225(59.21%) |
| Cough | 234(61.58%) |
| Dyspnea | 196(51.58%) |
| Myalgia | 179(47.11%) |
| Headache | 128(33.68%) |
| Chest pain | 81(21.32%) |
| Nausea | 101(26.58%) |
| Sputum | 83(21.84%) |
| Chills | 174(45.79%) |
| Hemoptysis | 13(3.42%) |
| Sore Throat | 15(3.95%) |
| Anorexia | 198(52.11%) |
| Rhinorrhea | 47(12.37%) |
| Anosmia | 54(14.21%) |
| Weakness | 207(54.47%) |
| Loss of consciousness | 22(5.79%) |
| Diarrhea | 70(18.42%) |
| Earache | 13(3.42%) |
| Wheezing | 0(0%) |
| Arthralgia | 31(8.16%) |
| Respiratory distress | 5(1.32%) |
| Dizziness | 63(16.58%) |
| Convulsion | 6(1.58%) |
| Abdominal pain | 32(8.42%) |
| Conjunctivitis | 23(6.05%) |
| Rash | 3(0.79%) |
| Skin lesion | 1(0.26%) |
| Lymphadenopathy | 0(0%) |
| Sweating | 19(5.00%) |
| Hematochezia | 2(0.53%) |
| Cold sweating | 14(3.68%) |
| **Venous blood gas analysis** | |
| WBC (10^9^/L) | 7.19±5.18 |
| Hemoglobin (g/dl) | 13.04±1.20 |
| Hematocrit (%) | 38.87±5.14 |
| Platelet (10^9^/L) | 194.42±83.32 |
| Lymphocyte (%) | 21.39±11.75 |
| Neutrophil (%) | 71.63±12.99 |
| *Lactate Dehydrogenase (LDH)* | 640.125±382.65 |
| **Complete blood count** | |
| pH | 7.41±0.1 |
| PO_2_ (mm Hg) | 35.19±19.42 |
| PCO_2_ (mm Hg) | 41.62±10.33 |
| HCO_3_ (mEq/L) | 26.31±7.10 |
| O_2_satVBG | 58.87±22.15 |
| **Kidney enzymes** | |
| Urea (mg/dL) | 47.40±38.33 |
| Creatinine (mg/dL) | 1.46±1.33 |
| **Others** | |
| Sodium (mEq/L) | 136.90±7.75 |
| CRP (mg/L) ^*^ | 53.00±48.54 |
| *Potassium (mEq/L)* | 4.06±0.68 |
| Calcium (mg/dL) | 8.27±1.08 |
| *Magnesium (mg/dL)* | 3.01±9.35 |
| *ESR (mm/hr)* ^*^ | 49.42±27.28 |
| *CPK (U/L)* ^*^ | 317.18±743.51 |
| *Blood sugar(mg/dL)* | 153.97±70.74 |
| *Bill Total* | 7.07±38.02 |
| *Procalcitonin (ng/ml)* | 1.05±1.88 |
| *PCR* ^*^ | 94(92.16%) |
| **Presenting vital sign** | |
| *Temperature (c)* | 36.91±3.31 |
| *Systolic BP (mmHg)* | 86.46±32.69 |
| *Diastolic BP (mmHg)* | 106.90±25.06 |
| *Respiratory rate (/min)* | 18.69±4.46 |
| *Heart rate (/min)* | 90.05±18.00 |
| *Saturation O_2_ (%)* | 92.15±7.67 |
| **Coagulation profile** | |
| *PT (s)*^*^ | 12.99±3.12 |
| *PTT (s)*^*^ | 28.85±11.86 |
| *INR (IU)*^*^ | 2.01±8.86 |
| **Liver enzymes** | |
| *AST (U/L)* ^*^ | 66.52±204.179 |
| *ALT (U/L)* ^*^ | 38.95±35.60 |

*Exposure to healthcare facilities (EHF)

history of traveling (HOT)

exposure to the suspected patient (ESP)

covid-19 infection in household (CIH)

Use of corticosteroid for comorbidities (UCC)

c-reactive protein (CRP)

erythrocyte sedimentation rate (ESR)

Creatine phosphokinase (CPK)

polymerase chain reaction (PCR)

Prothrombin time (PT)

Partial thromboplastin time (PTT)

international normalized ratio (INR)

aspartate aminotransferase (AST)

alanine transaminase (ALT)

# Appendix B

Figure B.1. The suggested set of 30 selected clinical labels from ExtraTree classifier.


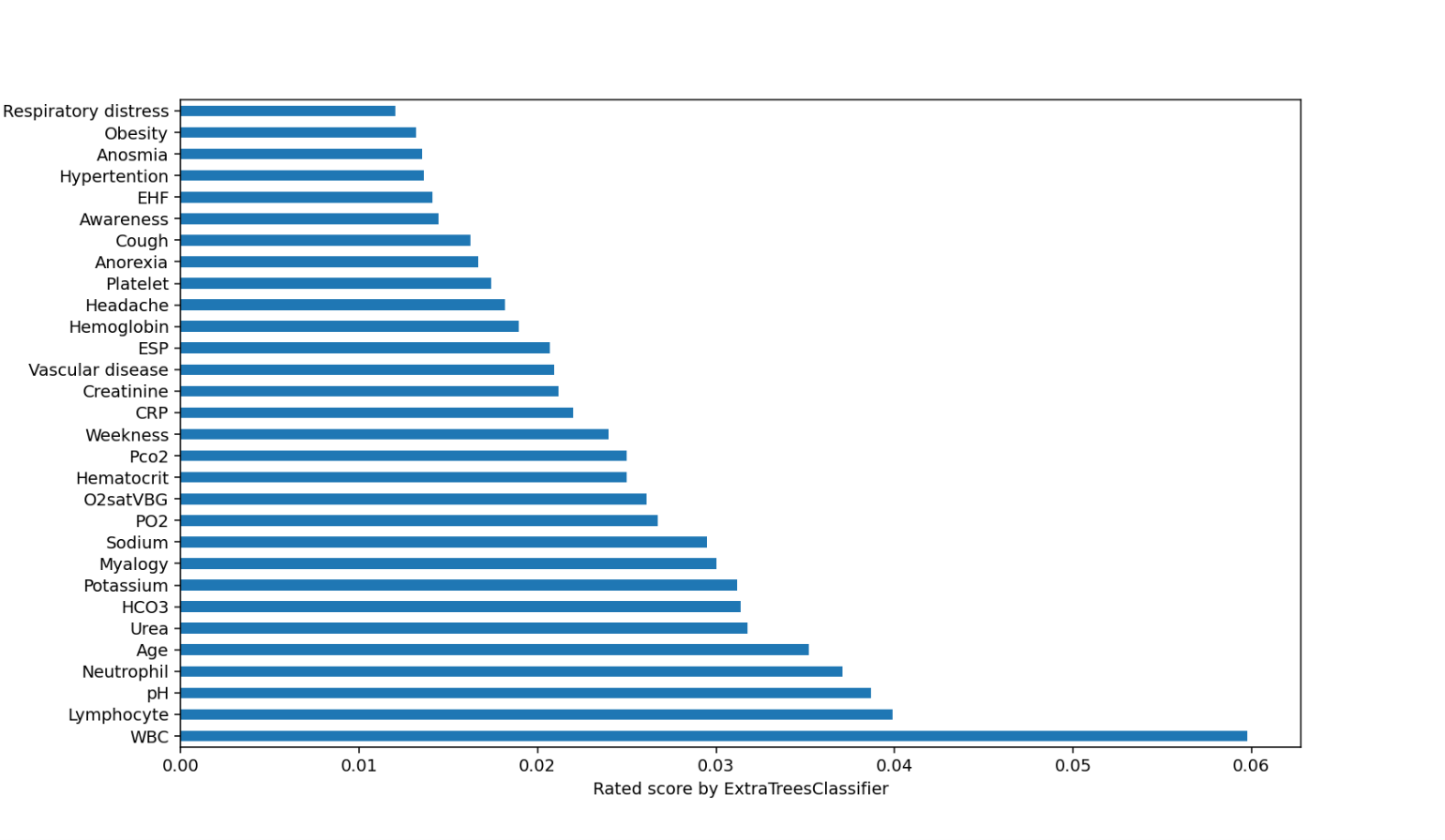


Figure B.2. The suggested set of 13 clinical labels from SelectKbest algorithm.


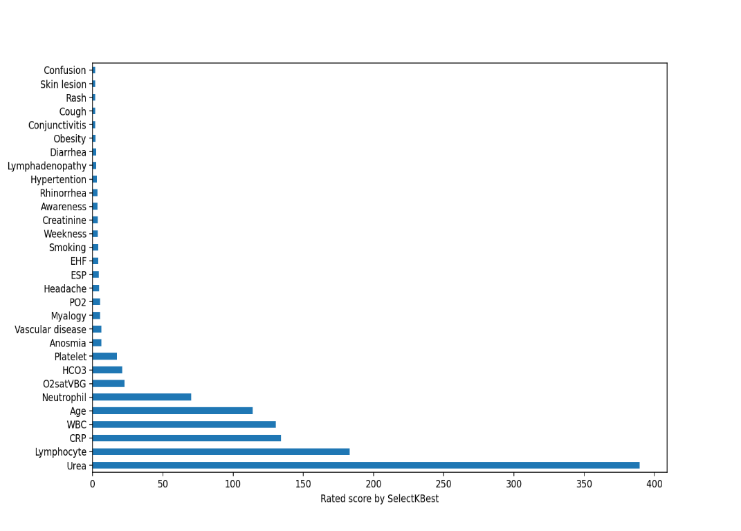

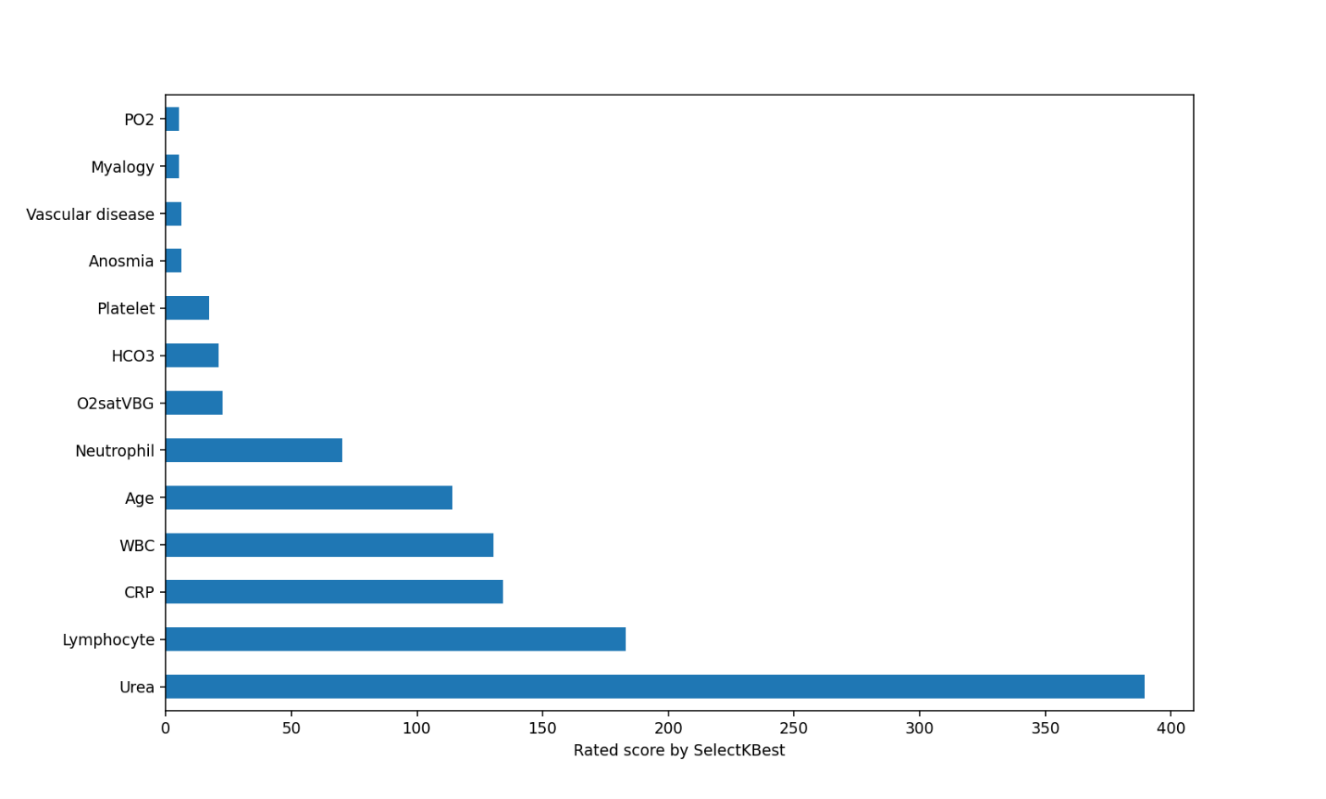


Set of 30 clinical labels from SelectKbest

# Appendix C

Table C.1. Selected hyperparameters from the Genetic Algorithm for the 3D-CNN CT model (section 3.3.2.1) and the medial 3D-CNN fusion model (section 3.3.3.1).

| **Hyperparameter for the** **3D-CNN CT model** | **Range** | **Hyperparameter for the medial 3D-CNN fusion model** | **Range** |
| --- | --- | --- | --- |
| Number of neurons of first layer | 32,64,128 | Number of neurons of first layer for CT images network | 32,64,128 |
| Number of neurons of second layer | 64,128,256 | Number of neurons of second layer for CT images network | 64,128,256 |
| Number of neurons of third layer | 128,256,512 | Number of neurons of third layer for CT images network | 128,256,512 |
| Drop out | round(uniform(0.1, 0.5), 1) | Drop out | round(uniform(0.1, 0.5), 1) |
| Optimization | Adamax, Adadelta, Adam,  Adagrad | Optimization | Adamax, Adadelta, Adam,  Adagrad |
| Learning rate | 0.000002, 0.000001 | Learning rate | 0.000002, 0.000001 |
| Number of layers | 1,2,3 | Number of layers  for CT images network | 1,2,3 |
| Kernel size | 3,5 | Kernel size | 2,4,8 |
|  |  | Number of neurons of first layer for clinical network | 67, 30, 15, 10 |
|  |  | Number of neurons of second layer for clinical network | 10, 8 |

# Appendix D

Table D.1. Results of the seven conventional algorithms in section 3.2.1
on the 67 original clinical labels.

| **Classifier** | **FPR** | **TPR** | **Recall^†^** | **Precision^†^** | **F0.5 score^†^** | **Kappa** | **Training accuracy** | **Test accuracy** |
| --- | --- | --- | --- | --- | --- | --- | --- | --- |
| SVM | 1 | 1 | 0.5 | 0.41 | 0.43 | 0 | 0.82 | 0.82 |
| MLP | 0.62 | 0.95 | 0.67 | 0.76 | 0.73 | 0.4 | 0.99 | 0.85 |
| KNN | 0.56 | 0.81 | 0.63 | 0.62 | 0.61 | 0.23 | 0.92 | 0.75 |
| Gaussian NB | 0.33 | 0.85 | 0.76 | 0.71 | 0.71 | 0.45 | 0.83 | 0.82 |
| XGBoost | 0.6 | 0.95 | 0.68 | 0.78 | 0.73 | 0.41 | 0.89 | 0.89 |
| Random Forest | 0.65 | 0.97 | 0.66 | 0.79 | 0.73 | 0.39 | 1 | 0.86 |
| Gradient Boosting | 0.54 | 0.92 | 0.69 | 0.72 | 0.71 | 0.39 | 0.97 | 0.97 |
| AVG | 0.61 | 0.92 | 0.65 | 0.68 | 0.66 | 0.32 | 0.92 | 0.85 |

**^†^** *Denotes the macro-averaging evaluation method*

Table D.2. Results of the seven conventional algorithms in section 3.2.1
on the 13 selected clinical labels from SelectKBest algorithm.

| **Classifier** | **FPR** | **TPR** | **Recall^†^** | **Precision^†^** | **F0.5 score^†^** | **Kappa** | **Training accuracy** | **Test accuracy** |
| --- | --- | --- | --- | --- | --- | --- | --- | --- |
| SVM | 1 | 1 | 0.5 | 0.41 | 0.43 | 0 | 0.82 | 0.82 |
| MLP | 0.57 | 0.92 | 0.68 | 0.71 | 0.7 | 0.37 | 0.94 | 0.83 |
| KNN | 0.58 | 0.81 | 0.62 | 0.61 | 0.6 | 0.21 | 0.92 | 0.74 |
| Gaussian NB | 0.47 | 0.89 | 0.71 | 0.71 | 0.71 | 0.41 | 0.85 | 0.83 |
| XGBoost | 0.6 | 0.96 | 0.68 | 0.81 | 0.75 | 0.42 | 1 | 0.86 |
| Random Forest | 0.67 | 0.96 | 0.65 | 0.81 | 0.71 | 0.35 | 1 | 0.85 |
| Gradient Boosting | 0.58 | 0.94 | 0.68 | 0.75 | 0.72 | 0.4 | 0.98 | 0.98 |
| AVG | 0.64 | 0.92 | 0.64 | 0.69 | 0.66 | 0.31 | 0.93 | 0.84 |

**^†^** *Denotes the macro-averaging evaluation method*

Table D.3. Results of the seven conventional algorithms in section 3.2.1
on the 30 selected clinical labels from ExtraTree classifier.

| **Classifier** | **FPR** | **TPR** | **Recall^†^** | **Precision^†^** | **F0.5 score^†^** | **Kappa** | **Training accuracy** | **Test accuracy** |
| --- | --- | --- | --- | --- | --- | --- | --- | --- |
| SVM | 1 | 1 | 0.5 | 0.41 | 0.43 | 0 | 0.82 | 0.82 |
| MLP | 0.53 | 0.92 | 0.7 | 0.75 | 0.72 | 0.42 | 0.94 | 0.84 |
| KNN | 0.54 | 0.81 | 0.64 | 0.63 | 0.62 | 0.24 | 0.92 | 0.75 |
| Gaussian NB | 0.37 | 0.89 | 0.76 | 0.75 | 0.75 | 0.51 | 0.86 | 0.83 |
| XGBoost | 0.56 | 0.96 | 0.7 | 0.8 | 0.76 | 0.46 | 1 | 0.87 |
| Random Forest | 0.67 | 0.97 | 0.65 | 0.83 | 0.73 | 0.37 | 1 | 0.86 |
| Gradient Boosting | 0.58 | 0.92 | 0.67 | 0.72 | 0.7 | 0.37 | 0.97 | 0.97 |
| AVG | 0.61 | 0.92 | 0.66 | 0.7 | 0.67 | 0.34 | 0.93 | 0.85 |

**^†^** *Denotes the macro-averaging evaluation method*

Table D.4. Results of the seven conventional algorithms in section 3.2.1
on the 25 extracted features from PCA algorithm.

| **Classifier** | **FPR** | **TPR** | **Recall^†^** | **Precision^†^** | **F0.5 score^†^** | **Kappa** | **Training accuracy** | **Test accuracy** |
| --- | --- | --- | --- | --- | --- | --- | --- | --- |
| SVM | 1 | 1 | 0.5 | 0.41 | 0.43 | 0 | 0.82 | 0.82 |
| MLP | 0.62 | 0.94 | 0.66 | 0.77 | 0.72 | 0.38 | 0.99 | 0.84 |
| KNN | 0.56 | 0.81 | 0.63 | 0.62 | 0.61 | 0.23 | 0.92 | 0.75 |
| Gaussian NB | 0.51 | 0.94 | 0.71 | 0.76 | 0.74 | 0.46 | 0.87 | 0.86 |
| XGBoost | 0.74 | 0.97 | 0.62 | 0.8 | 0.7 | 0.31 | 1 | 0.85 |
| Random Forest | 0.82 | 0.99 | 0.58 | 0.87 | 0.66 | 0.24 | 1 | 0.85 |
| Gradient Boosting | 0.77 | 0.95 | 0.59 | 0.69 | 0.64 | 0.22 | 1 | 0.82 |
| AVG | 0.72 | 0.94 | 0.61 | 0.7 | 0.64 | 0.26 | 0.94 | 0.83 |

**^†^** *Denotes the macro-averaging evaluation method*
